# Nutritional Genomics: Implications for Age-Related Macular Degeneration

**DOI:** 10.3390/nu16234124

**Published:** 2024-11-28

**Authors:** Inês Figueiredo, Cláudia Farinha, Patrícia Barreto, Rita Coimbra, Pedro Pereira, João Pedro Marques, Isabel Pires, Maria Luz Cachulo, Rufino Silva

**Affiliations:** 1 Ophthalmology Department, Unidade Local de Saúde Coimbra, 3004-561 Coimbra, Portugal; ines_500@hotmail.com (I.F.); claudia.farinha@hotmail.com (C.F.); pedro.n.pereira94@gmail.com (P.P.); marquesjoaopedro@gmail.com (J.P.M.); isabel.maravilha@sapo.pt (I.P.); mluzcachulo@gmail.com (M.L.C.); 2 AIBILI—Association for Innovation and Biomedical Research on Light and Image, 3000-548 Coimbra, Portugal; pbarreto@aibili.pt (P.B.); racoimbra@aibili.pt (R.C.); 3 Clinical Academic Center of Coimbra (CACC), 3000-548 Coimbra, Portugal; 4 Coimbra Institute for Clinical and Biomedical Research, Faculty of Medicine (iCBR-FMUC), University of Coimbra, 3004-531 Coimbra, Portugal

**Keywords:** age-related macular degeneration, dietary habits, micronutrients, personalized medicine

## Abstract

**Background:** Age-related macular degeneration (AMD) is a leading cause of vision loss in older individuals, driven by a multifactorial etiology involving genetic, environmental, and dietary factors. Nutritional genomics, which studies gene-nutrient interactions, has emerged as a promising field for AMD prevention and management. Genetic predispositions, such as variants in *CFH*, *C3*, *C2/CFB*, *APOE*, and oxidative stress pathways, significantly affect the risk and progression of AMD. **Methods:** This narrative review synthesizes findings from randomized controlled trials and recent advances in nutritional genomics research. It examines the interplay between genetic predispositions and dietary interventions, exploring how personalized nutritional strategies can optimize AMD management. **Results and Discussion:** The AREDS and AREDS2 trials demonstrated that supplements, including vitamins C, E, zinc, copper, lutein, and zeaxanthin, can reduce the progression to advanced AMD. Nutritional interventions tailored to genetic profiles show promise: *CFH* risk alleles may enhance zinc supplementation’s anti-inflammatory effects, while *APOE* variants influence the response to omega-3 fatty acids. Adjusting carotenoid intake, such as lutein and zeaxanthin, based on genetic susceptibility exemplifies emerging precision nutritional approaches. Ongoing research seeks to integrate nutrigenomic testing into clinical settings, enabling clinicians to tailor interventions to individual genetic profiles. **Conclusions:** Further studies are needed to assess the long-term effects of personalized interventions, investigate additional genetic variants, and develop tools for clinical implementation of nutrigenomics. Advancing these strategies holds the potential to improve patient outcomes, optimize AMD management, and pave the way for precision nutrition in ophthalmology.

## 1. Introduction

Age-related macular degeneration (AMD) is a neurodegenerative disease that affects the macula and is responsible for irreversible vision loss in individuals aged 55 and older [1]. It currently affects over 30 million people worldwide and is expected to increase tenfold by 2024 despite the introduction of new therapies for prevention and treatment [2,3].

AMD is commonly classified into three stages: early, intermediate, and late. In early AMD, small to medium-sized drusen are present [4]. Drusen are the earliest visible clinical sign of AMD [4]. Early in the disease process, lipids are deposited in Bruch’s membrane due to the retinal pigment epithelium’s (RPE) failure to process cellular debris associated with outer segment turnover [4]. This stage is typically asymptomatic and is managed with regular monitoring and lifestyle modifications, such as smoking cessation and a diet rich in antioxidants [5]. Intermediate AMD is characterized by larger drusen and pigmentary changes in the retinal pigment epithelium (RPE) [4,6]. Patients may experience mild visual disturbances, including difficulty adapting to low light or slight blurriness. Management at this stage often includes dietary supplementation to reduce the risk of progression to advanced stages [7]. Late AMD can manifest as either atrophic (dry) or neovascular (wet) macular degeneration [8]. Geographic atrophy (GA) is clinically recognized by a well-demarcated area of decreased retinal thickness. It is characterized by the loss of photoreceptors, RPE, and choriocapillaris, causing a gradual decrease in vision over time [9,10]. Wet or neovascular AMD is characterized by the ingrowth of new vessels from the choriocapillaris through a break in the Bruch´s membrane into the sub-RPE space [4,11]. These new vessels undergo plasma and blood extravasation, resulting in neurosensory or RPE detachment with fluid and blood accumulation [2,12]. In the end stage, neovascularization results in a fibrovascular or atrophic macular scar, with subsequent permanent damage to the central vision [4].

Several studies have investigated the factors associated with progression from non-advanced to advanced disease. This transition is multifactorial and influenced by demographic, lifestyle, phenotypic, and genetic factors, namely age, smoking, and nutrition [13].

### 1.1. Genetic Predispositions

AMD was the first human condition in which genome-wide association studies successfully identified genetic variants responsible for a substantial disease risk [14]. The genetic background for AMD has been well documented and is non-modifiable; 52 common AMD-associated variants and more than 100 rare variants have been reported, which explain most of the disease causes and helped identify several pathogenic pathways [1,15].

Numerous genetic risk variants have been linked to AMD, with particularly strong associations consistently identified for common variants at the *CFH* and *ARMS2*/*HTRA1* loci [15,16]. Genome-wide association studies (GWAS) have identified 52 variants across 34 genomic regions that are independently associated with AMD [17]. These findings have facilitated the development of a personalized genetic risk score (GRS) for the disease [15,17]. Additionally, GWAS revealed a significant presence of rare variants in the *CFH* and *CFI* genes [16,18]. Subsequent studies confirmed that these rare variants not only substantially increase disease risk but are also correlated with more severe phenotypes [15,19].

### 1.2. Environmental Factors

The gold standard treatment for the neovascular form is anti-Vascular Endothelial Growth Factor (anti-VEGF) injections. However, there is currently no approved treatment for GA, the predominant form of the disease [20]. Currently, we are limited to antioxidant/mineral supplementation, which might only delay disease progression [21].

Routine physical activity has been significantly associated with a decreased risk of AMD in several studies, even after adjustment for confounding factors such as age, body mass index, smoking habits, and dietary patterns like adherence to the mediterranean diet [21]. The exact mechanisms are not fully understood, but regular exercise is believed to enhance retinal health through improved systemic circulation, reduced oxidative stress, and modulation of chronic inflammation, all of which are implicated in AMD pathogenesis [1,22]. However, some researchers caution that a potential “healthy user bias” could contribute to these findings, as physically active individuals may engage in other health-promoting behaviors [21,23,24].

Smoking is one of the most significant modifiable risk factors for AMD, particularly for its advanced stages [15]. It increases the risk of late AMD by 2–4 times, with a weaker or negligible association with early AMD [25]. Smoking contributes to AMD through several mechanisms, including oxidative stress, chronic inflammation, impaired choroidal circulation, and the accumulation of retinal toxins [15,26]. Epidemiological evidence shows that smokers are more likely to develop severe AMD features such as larger drusen, RPE atrophy, and choroidal neovascularization [27,28]. While smoking cessation reduces AMD risk over time, former smokers still retain a higher risk compared to those who never smoked. Smoking also interacts with genetic predispositions, such as variants in the *CFH* and *ARMS2*/*HTRA1* loci, and compounds the risk through lower antioxidant levels, making dietary interventions particularly valuable [15]. Clinically, smoking cessation is a critical recommendation for AMD patients. Public health measures targeting smoking cessation could significantly reduce AMD incidence and slow disease progression.

### 1.3. Dietary Influences

Dietary interventions are gaining popularity in the treatment of several conditions. As an example, a 2022 study that explored the impact of a novel dietary supplement on sarcopenia tested a combination of leucine, omega-3 fatty acids, and the probiotic *Lactobacillus paracasei* PS23 over a two-month period [29]. Their results indicated the supplement’s potential in improving muscle health and function in sarcopenic individuals, highlighting its promise as a therapeutic dietary intervention [29].

Dietary intervention is emerging as a more cost-effective and broadly applicable method for AMD care [30]. Two landmark studies are notable on this topic. The first was the Age-Related Eye Disease Study (AREDS), a multicenter, randomized, placebo-controlled trial that evaluated the role of high-dose supplementation with zinc and antioxidants on the progression to advanced AMD [7]. This trial included 4757 participants, aged 55 to 80 years old, categorized into four AMD severity groups ranging from no AMD to advanced AMD in one eye. The follow-up lasted for an average of 6.3 years. Participants were randomly assigned to one of four treatment groups: antioxidants (vitamin C 500 mg, vitamin E 400 IU, and beta-carotene 15 mg), zinc (80 mg zinc oxide and 2 mg copper to prevent copper deficiency), antioxidants plus zinc, or placebo. Results found that: (1) the combination of antioxidants and zinc reduced the risk of progression to advanced AMD by 25% (*p* < 0.001) in individuals with intermediate AMD or advanced AMD in one eye; (2) the supplementation slowed the rate of vision loss, with a 19% reduction (*p* < 0.01) in the risk of significant visual acuity decline over 5 years; and (3) beta-carotene supplementation was associated with an increased risk of lung cancer in current or former smokers, highlighting the need for caution in these populations [7].

The second was the AREDS2 study, another multicenter, randomized, interventional trial, which aimed to refine the supplement formula by adding omega-3 fatty acids (DHA 350 mg, EPA 650 mg) and lutein/zeaxanthin (10 mg and 2 mg, respectively) while removing beta-carotene due to its risks in smokers [31]. AREDS2 included 4203 participants, aged 50 to 85 years old, with intermediate AMD in both eyes or advanced AMD in one eye. Participants were randomized into various treatment groups to assess the effects of the modified formulation vs. the original AREDS formula. The study duration was 5 years. Results from AREDS2 demonstrated: (1) replacing beta-carotene with lutein and zeaxanthin was equally effective in reducing AMD progression risk, with a 26% risk reduction (*p* < 0.001) for advanced AMD, being was safer for smokers and ex-smokers, (2) the addition of DHA and EPA did not have any statistically significant benefit in reducing AMD progression (*p* = 0.63), (3) reducing zinc dosage from 80 mg to 25 mg had no significant impact on AMD progression, suggesting flexibility in zinc dosing for safety concerns, (4) participants with low dietary intake of lutein and zeaxanthin had the greatest benefit from supplementation, with a 36% reduction (*p* < 0.001) in AMD progression when these carotenoids were added [31,32]. The AREDS2 formula is now the standard recommendation for managing intermediate or advanced AMD [31].

The AREDS studies showed that nutritional interventions can impact the rate of progression to advanced disease [31]. However, the long-term benefits and safety of using high doses of antioxidants, as might be required to prevent or slow AMD progression, have not been established [25,33]. There is limited evidence on whether well-nourished people with AMD need supplements or if a change to a particular type of diet further reduces the progression of AMD [34]. It has long been recognized that patients with the lowest intake of several nutrients face a higher risk of AMD vs. those with the highest intake [14]. This correlation is particularly evident for ω-3 fatty acids, especially docosahexaenoic acid (DHA), the carotenoids lutein and zeaxanthin, and varying degrees for zinc consumption [14,31,35]. However, these results are significantly influenced by differing populations, classification systems, and study designs [14]. Additionally, dietary patterns play a role in the risk of AMD. The Mediterranean diet is considered one of the most beneficial nutritional patterns regarding health benefits and has consistently been linked to greater longevity and a reduced risk of chronic age-related diseases [30]. Patients consuming diets with a higher glycemic index, meaning diets that deliver glucose to the bloodstream more rapidly, are also at an elevated risk for AMD [36,37]. Other dietary patterns, such as the Western diet, which includes high amounts of red meat, high-fat dairy products, processed meat, and refined grains, are linked to a significantly increased risk of AMD (Table 1) [14].

Exploring the connections between diet, micronutrients, and the host genome has opened new paths to treating several metabolic diseases. This review examined the interaction between diet, micronutrients, and genome in AMD, an area known as “nutritional genomics”. Adapting dietary interventions to reduce AMD risk can be refined by tailoring recommendations to an individual’s genetic profile, particularly for those with high-risk variants in genes such as *CFH* and *ARMS2*/*HTRA1*. By aligning diet with genetic vulnerabilities, it is possible to enhance the protective effects of nutrition and mitigate genetic risks.

For individuals with *CFH* variants, associated with complement activation and chronic inflammation, a diet rich in anti-inflammatory and antioxidant compounds is crucial [13,17,28]. These patients benefit from increasing the intake of foods high in lutein and zeaxanthin, such as spinach, kale, and egg yolks, as these carotenoids directly accumulate in the macula and reduce oxidative stress [31,33,38]. Additionally, consuming omega-3-rich foods like fatty fish (salmon, sardines) also helps counteract inflammation [39,40]. Polyphenol-rich foods, such as extra-virgin olive oil, berries, and green tea, can further modulate the complement system and inflammation [41]. Reducing dietary triggers of inflammation, such as trans fats and refined sugars, is equally important for individuals with *CFH*-related risks.

For patients with *ARMS2*/*HTRA1* variants, which are linked to mitochondrial dysfunction and increased susceptibility to drusen formation, enhancing mitochondrial support and reducing oxidative damage is key [15]. A diet emphasizing omega-3 fatty acids from fish or algal oils can strengthen mitochondrial function and protect photoreceptors from degeneration [42]. Foods rich in vitamin E, such as almonds, sunflower seeds, and avocados, provide lipid-soluble antioxidants that shield cellular membranes from oxidative damage [43]. In these individuals, avoiding diets high in saturated and trans fats, which exacerbate drusen accumulation and retinal stress, is critical.

Individuals with a combination of *CFH* and *ARMS2*/*HTRA1* variants, who are at the highest risk for AMD, may require a more comprehensive dietary strategy. Combining the benefits of the Mediterranean diet with targeted nutrient supplementation, such as the AREDS2 formulation (lutein, zeaxanthin, vitamin C, vitamin E, zinc, and copper), provides a robust approach to counteract their genetic risk. For these individuals, maintaining a consistent intake of these protective nutrients and avoiding detrimental dietary patterns, such as a high glycemic index (GI) diet, is especially important [31,36]. Low-GI foods like whole grains and legumes can help stabilize blood sugar levels, reducing oxidative stress and inflammation that might otherwise exacerbate genetic vulnerabilities.

## 2. Methods and Records: Selection Process

### 2.1. Study Design

This narrative review was conducted to evaluate the role of nutritional genomics in AMD. A comprehensive search of peer-reviewed literature was conducted using the PICO framework to identify relevant studies.

- Population (P): patients with AMD.
- Intervention (I): dietary factors, micronutrient supplementation, or genome-based personalized nutritional approaches.
- Comparison (C): standard care or differing personalized dietary interventions.
- Outcome (O): impact on AMD progression or prevention.

### 2.2. Search Strategy

A systematic search of electronic databases, including PubMed, Scopus, and Web of Science, was conducted from database inception to 10 October 2024. The search terms combined keywords and Medical Subject Headings (MeSH) terms, such as “age-related macular degeneration”, “diet”, “micronutrients”, “genome”, and “genetics”, using Boolean operators (AND, OR). Reference lists of eligible articles were also screened for additional relevant studies.

### 2.3. Inclusion Criteria

- Randomized controlled trials (RCTs), prospective and retrospective observational studies, experimental studies, and epidemiological studies.
- Articles published in English.
- Studies reporting on the association between micronutrients, dietary habits, genomic factors, and AMD.

### 2.4. Exclusion Criteria

- Studies not focused on AMD or without a dietary or genomic component.
- Reviews, conference abstracts, editorials, and articles not available in full text.
- High risk of bias based on the sum of randomization bias, missing data, outcome measurement bias, or selection bias.

### 2.5. Data Synthesis and Analysis

A narrative synthesis was employed to integrate findings from eligible studies. Results were categorized based on study design, key findings, and their relevance to AMD pathogenesis, management, and influence on progression. Divergent findings were discussed to highlight gaps in current knowledge and guide future research priorities.

**Figure 1:**
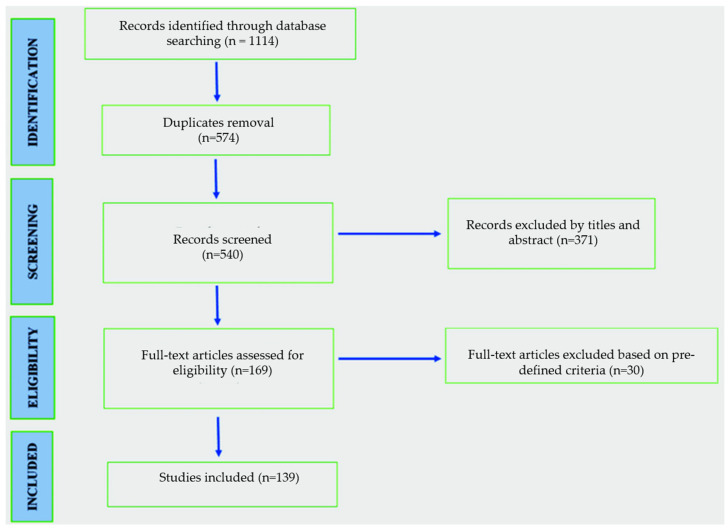
Flow diagram of screening studies.

A total of 1114 potentially relevant publications were identified in our initial search, along with manual reference screening (Figure 1). After duplicate publication removal and abstract review, 169 full-text articles were retrieved for a detailed evaluation. Following exclusion based on predefined criteria, 139 articles were included in the final analysis.

## 3. Genome and Mediterranean Diet in AMD

### 3.1. Genetic Risk Factors

Genetic factors play a crucial role in the development and progression of AMD, with genetic research significantly advancing our understanding of its underlying mechanisms [9]. Over 50% of the AMD heritability is attributable to common and rare variants across 34 loci, primarily associated with lipid metabolism, complement system, and extracellular matrix (ECM) restoration [17]. Understanding these genetic influences is crucial for advancing both diagnostic and therapeutic strategies for AMD.

#### 3.1.1. Complement Genes

The complement system, a critical part of the innate immune response, has been strongly implicated in AMD. Dysregulated complement activity leads to chronic inflammation and tissue damage, contributing to the development of both dry and wet forms of AMD [15,44]. Key genes in the complement cascade, including *CFH* (Complement Factor H), *C3* (Complement Component 3), and *C2*, have been associated with increased AMD risk.

*CFH* regulates complement activation and protects against excessive inflammation. In 2005, the *CFH* gene variant *rs1061170*, linked to the *rs570618* variant in the 2016 Genome-Wide Association Study (GWAS), was found to increase AMD risk and promote the progression to both neovascular AMD and GA [6,9]. The *Y402H* variant impairs *CFH* function, leading to uncontrolled complement activation and retinal damage, particularly in the macula [6]. Another *CFH* variant (*rs10922109*) has shown protective effects against late-stage AMD progression [45].

The *C3* gene is another critical player in complement activation. Intriguingly, the *C3 rs2230199* variant, while initially a susceptibility factor for GA development, appears protective against GA growth once the disease is established, suggesting that AMD-associated gene variants can display distinct effects at various stages [9,46].

Both *C2* and *CFB* gene variants have also been implicated in AMD, influencing complement activation and promoting inflammatory damage to the retina [9]. These genes act together in the alternative complement pathway, which is overactive in AMD patients. The minor allele *C2*/*CFB rs116503776* confers protection against late-stage AMD [45,46].

#### 3.1.2. ECM Restoration Genes

The ECM is a complex network of proteins and other molecules that provide structural and biochemical support to retinal cells, particularly the RPE [47,48]. Disruption in ECM remodeling contributes to the pathological features of AMD, such as drusen formation and GA. Several genetic loci related to ECM restoration have been identified as important for AMD susceptibility.

The *ARMS2* (Age-Related Maculopathy Susceptibility 2) gene is thought to influence oxidative stress and inflammation, both of which contribute to ECM degradation and the progression of retinal degeneration [9,49]. The *HTRA1* (High-Temperature Requirement A1) gene is involved in ECM remodeling and proteolysis [50,51]. Functional investigations appear to defend that *ARMS2* encodes a protein involved in mitochondrial function or extracellular processes, and *HTRA1* regulates TGF-β signaling, extracellular matrix deposition, and angiogenesis; their precise roles in AMD pathophysiology remain largely undefined [49,50].

The common *rs10490924* variant near the *ARMS2*/*HTRA1* genes significantly impacts AMD risk [9]. It is perfectly linked with *rs3750846* from the 2016 GWAS [17]. Prospective studies indicate that the minor allele increases the risk of early AMD, progression to neovascular AMD and GA, as well as GA growth rates of 0.23 mm/year for wildtype, 0.30 mm/year for heterozygous, and 0.32 for homozygous genotypes [52,53].

#### 3.1.3. Lipid Metabolism Genes

Lipid metabolism plays a critical role in retinal health, particularly in the accumulation and regulation of lipids in the retina and RPE [54]. Several genetic variants involved in lipid metabolism have been identified as risk factors for AMD, particularly those related to lipoprotein processing and cholesterol transport.

AMD-associated variants in these genes from the 2016 GWAS have been linked to AMD progression [17]. The *APOE* (Apolipoprotein E) gene, which regulates lipid metabolism, is one of the most studied genes in relation to AMD. Variants in the polymorphic *APOE* gene at amino acid positions 112 and 158 produce three isoforms: *ε2*, *ε3*, and *ε4*. While *APOE ε4* reduces AMD risk, *ε2* shows a trend toward increased risk [9,55]. These *APOE* isoforms and related variants have yet to be studied in AMD progression through prospective cohort studies [9].

Minor alleles of *rs2740488* in the *ABCA1* (ATP Binding Cassette Subfamily A Member 1) gene and *rs5817082* in the *CETP* (Cholesteryl Ester Transfer Protein) gene are protective against early AMD [9,46,52]. In the *LIPC* (Hepatic Lipase) gene, the minor allele of *rs2043085* is protective against late AMD [45,46].

### 3.2. Mediterranean Diet

Evaluating an overall dietary pattern, rather than isolated nutrient intake, is advantageous because nutrients may exhibit interactions and synergistic effects that exceed their individual impacts [56]. This is especially relevant in complex diseases like AMD, involving multiple pathways and risk factors [1,16].

The Mediterranean diet is characterized by a high intake of fruit, vegetables, cereals, fish, and olive oil; low-to-moderate consumption of dairy products; limited meat intake; and regular but moderate alcohol consumption, namely wine [21]. This dietary pattern is rich in antioxidants, unsaturated fats, lutein, and zeaxanthin, which are protective against AMD due to their antithrombotic and anti-inflammatory properties [41].

The Mediterranean diet has been widely associated with a decreased risk for AMD development and progression. Greater adherence to this diet has been linked to improved outcomes in cardiovascular and neurological diseases, which share risk factors and pathophysiological mechanisms with AMD, thus supporting its potential protective role in AMD [57,58]. In a 2023 report from the Coimbra Eye Study [1], low adherence to the Mediterranean diet was associated with an AMD risk 2.5 times higher. The combined effects of having a low adherence to the Mediterranean diet and a high genetic risk score resulted in a greater than 4-fold increased risk of AMD vs. a high adherence to the Mediterranean diet and a low genetic risk score [1].

The healthy retina’s metabolic activity is exceptionally high, requiring substantial energy for phototransduction and photoreceptor maintenance, leading to oxidative stress even under normal conditions [59]. The retina’s redox balance is crucial for its metabolic function; high oxidative stress can impair mitochondria, resulting in photoreceptor loss, cellular debris accumulation, and macular atrophy, all contributing to AMD [1]. Antioxidants, fatty acids, and carotenoids, such as lutein and zeaxanthin, support the structural integrity of the photoreceptor membrane and RPE cells and help maintain the antioxidant system at physiological levels [32,54].

## 4. Gene–Diet Interactions

### 4.1. Gene–Diet Interactions with Zinc

Zinc is an essential trace element involved in DNA synthesis, RNA transcription, cell division, survival, and immune system function, namely complement activation [9,35]. Cellular zinc levels are tightly controlled by transporters and proteins like metallothionein with antioxidant properties. Aging and oxidative stress reduce metallothionein levels in the macula, particularly in the fovea, releasing zinc into the extracellular compartment where drusen formation takes place [60]. Zinc (1) indirectly combats oxidative stress by stabilizing cell membranes and reducing lipid peroxidation; (2) supports RPE function by aiding the regeneration of photoreceptor pigments through its role in the enzyme retinol dehydrogenase; and (3) modulates the alternative complement pathway, potentially reducing chronic inflammation implicated in AMD pathogenesis [35]. As such, zinc intracellular loss can induce RPE and retinal cell apoptosis and increase oxidative damage, contributing to AMD development [9].

Zinc supplementation promotes zinc re-uptake into the RPE-choroid complex and enhances metallothionein synthesis [35]. However, systemic zinc levels are unreliable indicators of disease progression due to their variation in factors like age, sex, and fasting [61]. The AREDS1 study demonstrated that zinc supplementation (80 mg zinc oxide) lowered late AMD progression rates over six years [7]. In AREDS2, both high (80 mg) and low (25 mg) zinc doses showed similar benefits, suggesting lower doses could effectively slow AMD progression [31]. The Blue Mountains Study, a population-based cohort study that included 3654 participants aged 49 and older, suggested a protective effect of dietary zinc intake against early and overall AMD, with a potential threshold effect observed at intakes above 15.8 mg/day [62]. A 2014 study suggested that daily oral supplementation with 50 mg of zinc sulfate could reduce complement catabolism, as measured by the *C3d*/*C3* ratio, in AMD patients [35]. However, that effect appears limited to those with initially high complement catabolism. After the supplementation period ended, the *C3d*/*C3* ratio reverted to baseline, suggesting that zinc’s effect on complement activation is reversible [35]. Thus, ongoing zinc supplementation may be needed to sustain complement inhibition over extended periods.

The role of genotype in modulating response to zinc and antioxidant supplementation remains unclear [9,63]. Findings from the Rotterdam Study indicated that high dietary zinc intake may lower the risk of AMD associated with the *CFH* Y402H variant, suggesting a potential link between zinc intake and this genotype [64]. Additionally, a recent subgroup analysis using AREDS data found that responses to zinc and antioxidant treatments may be influenced by *CFH* and *ARMS2* genotypes, indicating that patients carrying the *CFH* Y402H risk allele might not benefit from zinc supplementation on the 10-year disease progression [63,65].

### 4.2. Gene–Diet Interactions with Vitamins

#### 4.2.1. Vitamin C and E

Vitamins C and E, two potent antioxidants, play fundamental roles in ocular health due to their protective effects against oxidative stress, a key contributor to AMD. Vitamin C, a water-soluble antioxidant, is present in high concentrations within the eye’s cornea, vitreous humor, and aqueous humor and functions as a cofactor in enzymatic processes [5]. Unlike some vitamins, vitamin C must be obtained from dietary sources, primarily fruits and vegetables, as humans cannot synthesize or produce it through gut microbiota [2]. Vitamin C neutralizes reactive oxygen species, regenerates vitamin E to its active form, and participates in collagen synthesis, which may stabilize Bruch´s membrane and reduce AMD-related structural degeneration [43].

Vitamin E, a lipid-soluble antioxidant, comprises tocopherols and tocotrienols, which integrate into cell membranes and neutralize free radicals, thereby preventing lipid peroxidation [2,43]. It also inhibits the activation of inflammatory mediators and reduces oxidative DNA damage in RPE cells [7].

Research linking vitamins C and E with AMD risk has yielded mixed results. Animal studies initially suggested that ascorbic acid (the physiologically active form of vitamin C) might protect the retina from oxidative damage, as ascorbate supplementation reduced retinal damage from light exposure [66]. Similarly, case–control studies involving neovascular AMD patients linked a high vitamin C and alpha-tocopherol intake with a reduced risk of AMD and a decreased risk of oxidative stress-related eye diseases [67,68]. These findings contrast with prospective studies in human populations, many of which have not found consistent associations. For instance, the Blue Mountains Eye Study observed that higher vitamin E intake was linked to increased late AMD risk, whereas the Eye Disease Case–Control Study found no significant link between vitamin E levels and AMD and reported that lower plasma levels of Vitamin C were associated with an increased risk of AMD [38,62].

In a study by Sant and colleagues, researchers examined ascorbate’s effect on DNA hydroxymethylation and gene expression in RPE cells [69]. VEGFA expression can be influenced by active DNA demethylation [69]. This process is initiated by TET (ten-eleven translocation) enzymes, which convert 5-methylcytosine (5mC) to 5-hydroxymethylcytosine (5hmC), an epigenetic marker that aids in regulating transcription [70]. TET enzymes require Fe^2^⁺ as a cofactor and 2-oxoglutarate (2OG) as a cosubstrate, and research shows that ascorbate enhances TET activity by promoting the conversion of 5mC to 5hmC [69]. In the absence of ascorbate, the hydroxylation reaction catalyzed by TET enzymes reaches a limit, and the oxidized iron (Fe^3^⁺ or Fe^4^⁺) becomes catalytically inactive. Ascorbate aids in regenerating active Fe^2^⁺ by reducing these oxidized species, thereby maintaining TET function [69]. However, oxidative stress, which contributes to AMD pathogenesis, oxidizes ascorbate to dehydroascorbic acid, depleting retinal ascorbate levels. Unlike ascorbate, dehydroascorbic acid cannot restore Fe^2^⁺, disrupting TET function and potentially altering gene transcription linked to AMD [71].

Human studies on vitamin E’s dietary impact on AMD reveal discrepancies. To date, only one study measuring systemic vitamin E levels over time suggested a possible protective effect [72]. The inconsistent findings across studies may result from variations in biological activity among the different tocopherol forms or differences in individual absorption rates. Further research is needed to understand better the precise role of vitamins C and E in AMD pathogenesis, particularly in light of their uncertain benefits in halting disease progression [2,9].

#### 4.2.2. Vitamin D

Vitamin D, primarily synthesized in the skin via ultraviolet B radiation as vitamin D3 (cholecalciferol) and secondarily obtained from dietary sources, especially oily fish, plays a vital role in calcium homeostasis, immune modulation, and insulin regulation [73,74]. The biologically active metabolite of vitamin D, 1,25-dihydroxyvitamin D, is distributed systemically and regulates these physiological processes. Circulating 25-hydroxyvitamin D (25(OH)D), which encompasses both D3 and D2, serves as the clinical measure of vitamin D status [74].

In European populations with low supplementation and limited food fortification, vitamin D deficiency, defined as 25(OH)D levels below recommended thresholds of at least 50 nmol/L, affects approximately 13% of individuals [75]. Genetic polymorphisms, mainly single nucleotide polymorphisms (SNPs) in genes regulating vitamin D uptake and metabolism, further influence serum 25(OH)D concentrations.

A recent review by Layana et al. highlighted several potential mechanisms through which vitamin D may influence AMD pathophysiology: (1) antioxidant protection against oxidative stress via suppression of pro-inflammatory cytokines released by macrophages and microglia (such as IL-6 and TNF-α) and upregulation of anti-inflammatory mediators; (2) possible inhibition of amyloid-beta deposits, which activate the complement cascade and inflammatory responses; and (3) anti-angiogenic effects through inhibition of hypoxia-inducible factor-1 (HIF-1) transcription, induction of endothelial cell apoptosis, and reduction in metalloproteinase (MMP)-9 production [76].

Studies investigating the relationship between 25(OH)D levels and AMD have shown inconsistent results. Many studies lacked sufficient power to examine late-stage AMD specifically or reported associations only within specific subgroups, such as by gender or age [77,78]. Some studies found that lower 25(OH)D levels were associated with early AMD, while others did not observe this link [79,80]. Additionally, findings on the association between specific SNPs influencing 25(OH)D levels and early or late AMD have been inconclusive [77]. A 2017 population-based, cross-sectional study found no linear association between 25(OH)D and early or late AMD or neovascular AMD. However, deficient vitamin D status (<25 nmol/L) was associated with neovascular AMD but with a small adjusted odds ratio; thus, the authors could not exclude residual confounding [74]. Significant associations with 25(OH)D were also found for SNPs in genes GC, VDR, CYP2R1, and CYP27B1: 2 SNPs (VDR) were associated with early AMD, 4 SNPs (RXRA), and 1 SNP (VDR) were associated with neovascular AMD, and 1 SNP (RXRA), 2 SNPs (VDR), and 1 SNP (CYP2R1) were associated with late AMD. After Bonferroni correction, however, no SNPs were associated with early AMD, late AMD, or neovascular AMD [74]. Therefore, there is still no clear evidence of an association of vitamin D pathway SNPs with AMD to date.

### 4.3. Gene–Diet Interactions with Carotenoids

#### 4.3.1. Carotenes

Carotenoids are organic pigments synthesized in plants, classified into carotenes (alpha-carotene, beta-carotene, and lycopene) and xanthophylls (lutein, zeaxanthin, and the isomer meso-zeaxanthin) [2,9]. Humans cannot biosynthesize carotenoids; thus, they must acquire them through dietary sources. Proposed mechanisms for carotenoid protection mechanisms against AMD include their antioxidative capacity, which involves activation of the Nrf2-ARE pathway to enhance the expression of antioxidant enzymes like glutathione peroxidase and catalase, and protection of RPE cells by reducing oxidative stress-induced apoptosis [9]. The primary carotenoids in the human diet are α- and β-carotene, lycopene, β-cryptoxanthin, lutein, and zeaxanthin.

Both α- and β-carotene act as precursors to vitamin A [9]. They are predominantly found in dark leafy vegetables (e.g., spinach, kale) and yellow/orange vegetables (e.g., carrots). Longitudinal reports have yielded inconsistent findings regarding the association between dietary levels of α-carotene and β-carotene and the onset or progression of AMD. While some studies report a protective effect, others find no significant correlation, and a few indicate a greater risk of late-stage AMD in individuals with higher consumption of β-carotene [62,67,81].

Studies suggest that carotenes interact with dietary patterns and genetic profiles to influence AMD risk. For example, individuals with high-risk genotypes (e.g., CFH, ARMS2) may benefit more significantly from carotene-rich diets [68,82]. Genetic variants in carotenoid metabolism genes (e.g., BCO1, BCMO1) affect β-carotene bioavailability and its subsequent genomic effects [25,82].

In AREDS1, β-carotene was included in the supplements to evaluate its effects on progression to central atrophy or exsudative AMD [7]. Five years later, 20% of cases receiving supplements progressed to late AMD, compared to 28% in the placebo group, with a significant decrease in risk observed only in individuals with intermediate-stage AMD or worse [7]. However, an increase in efficacy is observed when replacing beta-carotene with lutein/zeaxanthin in the original AREDS formulation [32]. High-dose β-carotene supplementation has been associated with an increased risk of lung cancer in smokers, raising concerns about its use in AMD populations where smoking prevalence is high [83]. Consequently, supplementation, according to AREDS1, is now recommended only for non-smoking patients with intermediate AMD.

#### 4.3.2. Xanthophylls

Xanthophylls, such as lutein, zeaxanthin, and meso-zeaxanthin, appear to be more readily released from food sources and are more efficiently transformed into micelles than carotenes like beta-carotene, enhancing their uptake by intestinal cells [84]. Lutein and zeaxanthin are present in the macular pigment located in ganglion cells, cone axons, and Müller cells and play a protective role by absorbing 40 to 90% of harmful blue and ultraviolet light [85]. The primary objective of the AREDS2 report was to enhance the original AREDS formulation by assessing the effects of lutein, zeaxanthin, eicosapentaenoic acid (EPA), and docosahexaenoic acid (DHA), while evaluating the impact of removing β-carotene and reducing zinc dosages [31]. No significant differences in progression to late AMD were observed between groups using the formula with lutein and zeaxanthin and those using the original formula. However, a subgroup analysis revealed a significantly lower progression to late AMD among those receiving lutein and zeaxanthin without β-carotene [9,31].

Evidence linking lutein and zeaxanthin supplementation to increased systemic levels has been documented, but findings regarding the relationship between systemic concentrations and macular pigment optical density have been inconsistent [86,87,88]. Longitudinal studies examining dietary lutein and zeaxanthin’s impact on AMD progression have produced mixed results. While some confirm a protective effect against early AMD development and progression to late-stage AMD, others find no association, and one report showed an opposite effect [62,81,89]. A recent post hoc analysis from the AREDS and its follow-up AREDS2 showed that oral antioxidant supplementation may decelerate the progression of GA in late-stage AMD, particularly noncentral GA [90]. The study revealed that antioxidant supplements had limited effects once GA reached the fovea; however, for patients with noncentral GA, where the fovea remains unaffected (“foveal sparing”), supplements reduced GA expansion towards the fovea by approximately 55% over 3 years [90]. Specifically, in AREDS participants with noncentral GA, the progression rate towards the fovea was slower in those randomized to antioxidants (50.7 μm/year) than those without (72.9 μm/year, *p* = 0.012). Similarly, in AREDS2 participants with noncentral GA randomized to lutein/zeaxanthin, progression slowed to 80.1 μm/year vs. 114.4 μm/year in those without supplementation (*p* = 0.011) [90]. These findings support the continued use of AREDS2 supplements for patients with late-stage dry AMD to slow central vision loss, as antioxidants appear to enhance the natural foveal-sparing effect, preserving central vision, which is vital for tasks such as reading and driving.

One possible explanation for the inconsistencies observed in previous studies may lie in the varying responses of macular pigment to dietary intake of macular carotenoids [89,91,92]. While lutein and zeaxanthin are obtained exclusively from dietary sources or supplements, their subsequent accumulation in the retina is influenced by multiple factors, including various genetic components [91,92]. Recent findings from a twin study indicate that approximately 27% of the macular response to dietary carotenoids is inherited, and research supports that genetic variations significantly determine carotenoid levels in the retina and serum [82,91,93]. Additionally, the relationships between dietary or serum carotenoids and AMD may reflect other unknown factors related to diet and lifestyle that were not accounted for, while genetic indicators of carotenoid status would not be subject to such confounding influences.

Lutein inhibits the activation of STAT3 by inflammatory cytokines and extracellular signal-regulated kinase (ERK), thereby reducing DNA damage and maintaining a-wave electroretinogram (ERG) amplitude in a study with mouse models [94]. Additionally, lutein diminishes the expression of hypoxia-inducible factor 1α, suppresses the production of reactive oxygen species, and lowers VEGF expression [95].

The 2014 study by Meyers and colleagues tested variants in genes related to lutein and zeaxanthin status for association with AMD in the Carotenoids in the Age-Related Eye Disease Study (CAREDS) [96]. The genetic variants associated with AMD were located in genes involved in (1) cholesterol and carotenoid membrane transport proteins in the intestine and retina (SCARB1, NPCL1L1, and ABCA1) and/or high-density lipoprotein levels in the bloodstream (SCARB1, APOE, and ABCA1); (2) carotenoid cleavage enzymes (BCMO1 and BCO2); (3) omega-3 fatty acid status (FADS2); and (4) an inherited retinopathy characterized by the complete absence of macular pigment (ALDH3A2) [96]. The authors found that a total of 24 variants across five genes that had not been previously linked to AMD (BCMO1, BCO2, NPCL1L1, ABCG8, and FADS2), along with four genes known to be associated with AMD (SCARB1, ABCA1, APOE, and ALDH3A2), were independently associated with AMD after adjusting for age and ancestry [96]. Variants in all these genes—though not always in the same SNPs—were associated with serum and/or macular levels of lutein and zeaxanthin. A genetic risk score incorporating nine variants significantly distinguished between AMD cases and controls, independent of age, smoking status, CFH Y402H, and ARMS2 A69S. The odds ratio for AMD among women in the highest quintile of the risk score compared to those in the lowest was 3.1 [96].

### 4.4. Gene–Diet Interactions with Lipids

#### 4.4.1. Cholesterol and Triglycerides

Cholesterol is an organic compound synthesized by cells or obtained through diet. It is a vital structural element of cellular membranes, participates in signaling pathways, and acts as a precursor for synthesizing steroid hormones, bile acids, and vitamin D [9]. Most studies have not found a significant correlation between systemic cholesterolemia and the progression to early-stage AMD, GA, or neovascular AMD [22,97]. One study, however, found that higher serum cholesterolemia was protective against neovascular disease while simultaneously posing a risk for the onset of GA over a five-year follow-up period [26].

Triglycerides consist of glycerol esterified to three fatty acids of varying lengths. They are the main components of body adipose tissue and are present in the bloodstream, facilitating the transport of fat and glucose [9]. Most reports have not found a relationship between systemic triglyceridemia and progression of AMD [24,98,99]. One prospective study reported a lower risk of developing AMD after an 18-year follow-up for individuals with higher baseline systemic triglyceridemia [100].

#### 4.4.2. Fatty Acids

Fatty acids comprise a hydrocarbon chain, a carboxyl, and a methyl group at each end [54]. They are involved in energy storage as triglycerides, forming a significant component of the lipid bilayer in cellular membranes and contributing to the formation of cholesterol esters [54]. Fatty acids are classified based on the number of carbon atoms and the presence of double bonds as follows:

- Saturated fatty acids (SFAs) without double bonds;
- Monounsaturated fatty acids (MUFAs) with one double bond;
- Polyunsaturated fatty acids (PUFAs) with at least two double bonds [54]. PUFAs are further categorized into two families: omega-3 and omega-6, based on whether the first double bond occurs at the third or sixth carbon from the terminal methyl group [54].

Most epidemiological studies indicate that high consumption of SFAs and trans-SFAs may be linked to an increased risk of AMD [39,40,79,101]. However, many of these findings lack statistical significance, preventing definitive conclusions about fatty acids as hazardous for AMD. Trans-SFAs are structurally rigid compared to cis-unsaturated fatty acids. They compete with omega-3 (e.g., DHA) and omega-6 fatty acids for incorporation into cell membranes and enzymatic pathways, reducing the bioavailability of DHA, which is a critical component of the photoreceptor outer segment [64,102]. When incorporated into retinal cell membranes of RPE and photoreceptors, they reduce membrane fluidity, disrupt the function of membrane-bound proteins and nutrient transporters, impair cellular signaling pathways critical for retinal health, and stimulate the production of inflammatory cytokines (e.g., IL-6, TNF-α) and chemokines (e.g., MCP-1) via activation of nuclear factor-κB (NF-κB).

The relationship between MUFAs and AMD is inconsistent across various epidemiological studies [39,42,103]. Few showed significant findings, making the evidence for MUFAs’ potential role in AMD prevention unconvincing [54]. Among MUFAs, oleic acid has been suggested to offer protective effects against AMD, though research on its direct association is limited. Studies from Australia and France have linked olive oil consumption—rich in oleic acid—to a lower risk of AMD [42,104]. However, olive oil has other beneficial compounds, namely polyphenols, complicating the determination of whether the protective effects stem from oleic acid or these additional components [54]. MUFAs enhance retinal cell membrane fluidity and stability, supporting photoreceptor and RPE function and generating less lipid peroxidation, thereby reducing oxidative damage [13,103]. They exhibit anti-inflammatory effects by suppressing NF-κB activity and also promote cholesterol efflux via HDL, reducing lipid accumulation and drusen formation [101]. These properties collectively support retinal health and reduce AMD risk.

Omega-3 LC-PUFAs, with over 18 carbon atoms, are essential fats humans cannot synthesize. Alpha-linolenic acid (ALA) can be converted into other omega-3 LC-PUFAs, like EPA and DHA, but this conversion is inefficient in humans [105]. Algae represent the main sources of EPA and DHA, which are then consumed by fish, making them rich in these fatty acids [54]. Omega-3 LC-PUFAs play crucial roles in the structure and protection of the retina, with DHA being particularly concentrated in photoreceptor membranes. EPA and DHA also possess anti-inflammatory properties by suppressing inflammation via inhibition of NF-κB and reduction in pro-inflammatory cytokines and promoting anti-inflammatory lipid mediators like resolvins and neuroprotectins [44]. Additionally, omega-3 LC-PUFAs may enhance the density of macular pigment, providing blue light filtering and antioxidant benefits [40,102,106].

A 2008 meta-analysis combining various studies found that greater intake of omega-3 PUFAs resulted in a 38% lower likelihood of AMD [42]. Similarly, high fish consumption was associated with a 33% reduced risk [42]. Two randomized controlled trials, AREDS2 and NAT-2, examined the impact of omega-3 supplementation on AMD. AREDS2 did not find a significant reduction in AMD risk with EPA and DHA supplementation [31]. However, NAT-2 suggested that patients with the highest blood omega-3 concentration had a smaller risk of neovascularization [107,108]. It is worth noting, however, that both formulations were different; the AREDS2 formulation was composed of ethyl-esters with a 1:2 DHA/EPA ratio, and the NAT-2 formulation included triglyceride with a 3:1 DHA/EPA ratio, with consequent differences in bioavailability [54]. Both studies highlight the need to further explore omega-3’s potential protective roles in AMD.

A 2011 study examined the effects of dietary omega-3 long-chain PUFA (ω3s) and reduced linoleic acid (LA) intake on the neurosensory retina and RPE in rats [109]. Diets high in ω3s significantly enhanced their incorporation into various tissues, especially when LA intake was low. Low LA diets increased LDL-receptor gene expression and showed trends in upregulating lipid metabolism-related genes, though retinal function remained unaffected [109]. The findings suggest that increasing ω3 intake while lowering LA may support the enrichment of ω3s in retinal tissues and have a preventive role against AMD [109].

Omega-6 PUFAs, particularly linoleic acid, have been linked to inflammation, which may contribute to retinal damage and the development of AMD [110]. Arachidonic acid, in particular, contributes to AMD through pro-inflammatory pathways by generating eicosanoids like prostaglandins and leukotrienes, which exacerbate retinal inflammation [105]. Epidemiological evidence suggests that higher intake of omega-6 PUFAs correlates with an increased risk of AMD, although significant associations are less frequently reported [39,86,111]. Excessive omega-6 intake relative to omega-3 may disrupt lipid homeostasis, promote oxidative stress, and increase VEGF expression, and this imbalance may accelerate AMD progression [111].

In summary, omega-3 LC-PUFAs are beneficial for retinal health and may protect against AMD, while omega-6 PUFAs could exacerbate risk through inflammatory pathways. Further research is needed to clarify these relationships and optimize dietary recommendations for AMD prevention.

#### 4.4.3. Lipoproteins

Lipids like cholesterol and triglycerides are essential for energy storage, cell signaling, and forming cell membranes. However, because they are insoluble in water, they require transport vehicles called lipoproteins to circulate in the bloodstream [112]. Lipoproteins vary in composition and properties and are classified into five categories based on density.

Total cholesterol (TC) is the most commonly investigated lipoprotein related to AMD, which has shown a weak or insignificant association with AMD in most studies [47,54]. However, a recent meta-analysis indicated a protective tendency of higher TC levels in early AMD [113]. Triglycerides (TG), primarily a fat source, have similarly shown mixed results. While many studies found no significant link to AMD, some noted lower TG levels in AMD patients and a potential inverse relationship, particularly in the early stages [47,113].

Low-density lipoprotein cholesterol (LDL-C) is often associated with increased cardiovascular disease risk but showed mixed results in AMD studies [47,113]. LDL-C contributes to AMD by accumulating in the Bruch’s membrane and sub-retinal space, where it undergoes oxidative modification [113]. Oxidized LDL triggers chronic inflammation via the activation of macrophages and complement pathways, promoting drusen formation. Some smaller studies found higher LDL-C amounts in AMD patients, while larger populational cohorts did not confirm these findings [47,54]. Most LDL-C measurements are calculated rather than directly measured, highlighting a need for more accurate assessments.

High-density lipoprotein cholesterol (HDL-C) results are also inconsistent. A review of multiple studies found no association in the majority, with some indicating higher or lower levels in AMD patients [47,113]. A meta-analysis suggested a possible increased risk for AMD in patients with greater HDL-C levels [113]. Recent genetic studies support that elevated HDL-C might increase AMD risk, prompting a focus on HDL functionality over mere levels [114]. Research indicates that inflammation and oxidative stress can alter HDL, converting it into dysfunctional particles [115]. One study noted significant increases in serum amyloid A in AMD patients, while other HDL components, such as apolipoproteins A-I (APOA1), A-II (APOA2), *C*-III, E (APOE), TG, phospholipids, fatty acids of HDL, and total and non-esterified cholesterol, showed no difference compared to controls [116]. Moreover, HDL’s role in complement regulation—linked to AMD pathogenesis—suggests that lipoproteins might influence AMD development through inflammatory processes [18,23].

Over the past decade, numerous genetic variants linked to lipid levels have been identified. The first large GWAS in this area, with over 100,000 participants, identified 95 loci that collectively account for 25–30% of the genetic variance in these traits [117]. Subsequent studies expanded the sample to 188,577 patients, revealing an extra 62 loci associated with lipid levels [118]. Advancements in reference panels, such as the 1000 Genomes Project, have identified even more loci and finer mapping of known variants [119]. Many of these loci are also implicated in metabolic disorders, namely coronary artery disease and diabetes [118]. Recent techniques like whole-exome sequencing have uncovered rare variants with more significant effects, primarily within already identified loci [120].

Key variants related to lipid metabolism include those close to genes *LIPC*, *CETP*, *ABCA1*, and *APOE* [17].

Plasma HDL concentrations are modulated by *LIPC*, which is localized within the subretinal space and involved in the intra-retinal transport of lipids [121]. Multiple investigations have identified a protective association between specific *LIPC* polymorphisms (*rs493258*, *rs10468017*, *rs9621532*, *rs11755724*, *rs509859*, and *rs12637095*) and the development of AMD [122,123]. These variants’ mechanisms for reducing AMD risk remain ambiguous [124]. In fact, increased HDL levels might be correlated with a heightened risk of AMD [15]. It has been hypothesized that these genetic variants may enhance the HDL-facilitated delivery efficiency of carotenoids to the retina [125]. Conversely, the rare LIPC variants *rs13095226* and *rs3748391* have been associated with a modestly elevated risk for AMD [15]. Lastly, specific LIPC variants may correlate with diminished efficacy in response to anti-VEGF therapies [126].

APOE, a key component of HDL in the retina, facilitates cholesterol and lipid efflux from photoreceptor cells and the RPE via interaction with receptors like LDLR (low-density lipoprotein receptor) and LRP1 (LDL receptor-related protein 1) [55]. Disrupted APOE-mediated lipid clearance leads to lipid accumulation, drusen formation, and retinal dysfunction. The *APOE* gene is a significant genetic risk factor for AMD [127]. The *APOE2* allele is associated with an increased risk of AMD, while *APOE4* appears to reduce risk compared to the wildtype *APOE3* by altering lipoprotein binding properties [20]. Interestingly, *APOE4* is also the primary genetic risk factor for Alzheimer’s disease, a condition that shares characteristics such as neuroinflammation and amyloid-beta deposition with AMD [20]. Poor clearance of amyloid-beta contributes to drusen composition and pro-inflammatory signaling in AMD [55,127].

*CETP*, which facilitates reverse cholesterol transport, and *ABCA1*, which is involved in cholesterol efflux, are also linked to both conditions. Variants in *CETP* and *ABCA1* correlate with HDL-C and TC levels and have been associated with AMD risk [117,128]. *CETP* inhibitors have been exploited for cardiovascular conditions, with notable increases in HDL-C levels observed in clinical trials [129]. However, given that *CETP* variants linked to high HDL-C levels are associated with increased AMD risk, using *CETP* inhibitor drugs to raise circulating HDL-C may not be a viable strategy for AMD treatment [54]. A study explored the *APOA1* mimetic peptide 4F as a local AMD treatment [130]. This synthetic peptide mimics the anti-atherogenic properties of APOA1, binding to oxidized lipids and reducing atherosclerotic lesions in animal models [131]. Phase II trials showed that 4F improved the HDL anti-inflammatory index, but no enhancement in HDL functional biomarkers were seen, and it was well tolerated [132]. In Apoe-null mice, 4F injections decreased esterified cholesterol in Bruch’s membrane and improved its structure, suggesting it could effectively target lipid deposits in AMD [130]. Currently, no drugs targeting *CETP* or *LIPC* expression have been approved for clinical use [54]. Thus, new therapies targeting HDL-C metabolism for AMD are still in their early stages and must proceed cautiously to avoid increasing cardiovascular risk, where HDL-C is typically protective [54].

*VEGF*, a key player in angiogenesis, is similarly connected to lipid metabolism and AMD. Although some variants associated with lipid levels are also linked to AMD, further research is needed to clarify these relationships and the specific roles of these genetic factors in both conditions [133].

Mitochondrial dysfunction in AMD resembles the Warburg effect, characterized by decreased mitochondrial activity and a shift to anaerobic glycolysis [134]. Peroxisome proliferator-activated receptor γ coactivator 1α (PGC-1α), a key regulator of mitochondrial biogenesis and lipid metabolism, is known to enhance fatty acid oxidation and mitochondrial function [135,136]. A 2024 research by Zhou and colleagues found that inhibiting PGC-1α—even on a regular diet—can induce RPE dysfunction and drusen-like deposits in mice [137]. This inhibition leads to reduced expression of lipid metabolism genes, decreased mitochondrial mass and function, and increased susceptibility to oxidative stress, highlighting the role of PGC-1α in lipid metabolism within the RPE and suggesting new avenues for potential AMD treatments [137].

The association of genetic variants in HDL-C metabolism genes advances the potential for pharmacological strategies targeting HDL-C in treating AMD [54]. However, several critical questions remain before advancing these therapies. First, it is unclear whether pharmacological interventions should aim to raise or lower HDL-C levels. Mendelian Randomization investigations have produced conflicting results regarding the relationship between CETP and LIPC variants and AMD risk [114,138]. Second, determining whether local or systemic HDL-C is involved in AMD is essential for developing effective pharmacological interventions. Oral treatments may be appropriate for managing intermediate AMD, while advanced stages may require more direct methods, such as intravitreal injections [54].

A 2021 study also suggested that diet could influence the retinal transcriptome even in the absence of the gut microbiome. High-throughput RNA sequencing revealed that a high-fat diet affected the expression of genes and pathways related to retinal inflammation, angiogenesis, and RPE function, regardless of gut microbiome presence [139]. These findings suggest that intricate interactions between diet, microbiome, and retinal health are only now being acknowledged as significant in both retinal physiology and the development of retinal diseases [139].

## 5. Conclusions and Future Perspectives

Nutritional genomics offers promising insights into the prevention and management of AMD by highlighting the interplay between diet, genetic susceptibility, and retinal health. Research has shown that specific dietary patterns can positively influence and modulate gene expression associated with AMD. These findings underscore the potential for targeted nutritional interventions to mitigate the risk of AMD, especially in genetically predisposed individuals. As our understanding of the genetic factors influencing AMD deepens, integrating personalized nutrition strategies may enhance protective effects against this leading cause of blindness.

Future treatment perspectives for AMD could focus on developing tailored dietary guidelines and supplements that align with individual genetic profiles. Clinical trials exploring the efficacy of omega-3-rich diets and other bioactive compounds will be essential in establishing concrete recommendations for AMD prevention and management. Additionally, combining nutritional approaches with emerging pharmacological therapies may create synergistic effects, leading to more effective interventions. Key areas for future research avenues in nutritional genomics related to AMD include: (1) Long-term Effects of Personalized Dietary Interventions to investigate how tailored diets based on genetic predispositions affect AMD progression over decades. Specific areas to explore include the adaptation of AREDS supplements for genetic variants such as *CFH* and *ARMS2*, the relationship between genotype-specific dietary modifications and macular pigment density, and the identification of biomarkers in response to dietary changes; (2) exploration of novel genetic variants beyond *CFH* and *ARMS2*, to identify additional genetic loci influencing dietary response and AMD progression. This includes studying epigenetic changes (e.g., methylation and histone modifications), examining the role of less-studied variants involved in lipid metabolism (e.g., *APOE*, *LIPC*) and oxidative stress (e.g., *SOD2*), and developing polygenic risk scores that integrate genetic, dietary, and lifestyle factors to predict AMD risk and response to interventions; (3) exploration of gene–diet interactions in AMD across diverse populations to understand ethnic and regional differences, for example, the impact of *Vitamin D receptor gene* (*VDR*) variations in AMD risk in populations with differing sunlight exposure and dietary vitamin D levels; and (4) examination of the protective effects of antioxidants beyond AREDS in AMD prevention and treatment, such as the impact of dietary flavonoids (e.g., from green tea or berries) on retinal health and the potential role of vitamin K2 in maintaining retinal microvasculature integrity.

Ultimately, incorporating nutritional genomics into clinical practice can revolutionize AMD treatment strategies, fostering a more holistic approach to preserving vision and retinal health as the population ages.

## Figures and Tables

**Table 1:** Comparative analysis of Mediterranean diet vs. Western diet and implication on AMD risk.

	Mediterranean Diet	Western Diet
Definition	Diet rich in fruits, vegetables, whole grains, nuts, legumes, olive oil, moderate fish and poultry, with low consumption of red meat and dairy.	Diet high in processed foods, red and processed meats, refined sugars, saturated fats, and low in fresh produce.
Key nutrients	High levels of antioxidants (vitamins C and E, lutein, zeaxanthin), omega-3 fatty acids (DHA, EPA), and polyphenols.	High intake of saturated fats, refined carbohydrates, and low levels of protective nutrients like omega-3 and antioxidants.
Impact on AMD risk	Associated with a 30–40% lower risk of AMD progression and development due to anti-inflammatory and antioxidant properties.	Associated with a higher risk of AMD development and faster progression to advanced stages.
Oxidative Stress	Reduce oxidative damage to the retina, a key factor in AMD pathogenesis.	Promotes oxidative stress due to low antioxidant intake and high pro-oxidant components.
Inflammation	Anti-inflammatory effects mediated by omega-3s, polyphenols, and monounsaturated fats reducing chronic retinal inflammation.	High levels of pro-inflammatory fats and refined sugars exacerbate chronic inflammation in the retina.
Cardiovascular impact	Improves cardiovascular health, indirectly benefiting retinal vasculature.	Poor cardiovascular health contributes to retinal damage.
Genetic Interactions	Protective effects mitigate genetic risks associated with *CFH*, *ARMS2***/***HTRA1*, and other AMD-associated variants, reducing the influence of high-risk alleles.	Amplifies the effects of AMD risk variants, particularly in *CFH* and *ARMS2/HTRA1*, through increased inflammation and oxidative stress.
*CFH* gene	Reduces AMD risk in individuals with *CFH* variants, as antioxidants lower complement activation.	Exacerbates AMD risk in individuals with *CFH* variants by promoting complement system dysregulation.
*ARMS2*/*HTRA1* Genes	Anti-inflammatory properties counteract risks associated with *ARMS2*/*HTRA1*, slowing drusen accumulation and AMD progression.	High-fat Western diets accelerate drusen deposition and choroidal neovascularization in individuals with *ARMS2*/*HTRA1* variants.

## References

[1] Barreto P., Farinha C., Coimbra R., Cachulo M.L., Melo J.B., Lechanteur Y., Hoyng C.B., Cunha-Vaz J., Silva R. (2023). Interaction between Genetics and the Adherence to the Mediterranean Diet: The Risk for Age-Related Macular Degeneration. Coimbra Eye Study Report 8. Eye Vis..

[2] Rinninella E., Mele M.C., Merendino N., Cintoni M., Anselmi G., Caporossi A., Gasbarrini A., Minnella A.M. (2018). The Role of Diet, Micronutrients and the Gut Microbiota in Age-Related Macular Degeneration: New Perspectives from the Gut−Retina Axis. Nutrients.

[3] Wong W.L., Su X., Li X., Cheung C.M.G., Klein R., Cheng C.-Y., Wong T.Y. (2014). Global Prevalence of Age-Related Macular Degeneration and Disease Burden Projection for 2020 and 2040: A Systematic Review and Meta-Analysis. Lancet Glob. Health.

[4] Gheorghe A., Mahdi L., Musat O. (2015). Age-Related Macular Degeneration. Rom. J. Ophthalmol..

[5] Ambati J., Fowler B.J. (2012). Mechanisms of Age-Related Macular Degeneration. Neuron.

[6] Seddon J.M., Reynolds R., Rosner B. (2009). Peripheral Retinal Drusen and Reticular Pigment: Association with CFHY402H and CFHrs1410996 Genotypes in Family and Twin Studies. Investig. Ophthalmol. Vis. Sci..

[7] Age-Related Eye Disease Study Research Group (2001). A Randomized, Placebo-Controlled, Clinical Trial of High-Dose Supplementation with Vitamins C and E, Beta Carotene, and Zinc for Age-Related Macular Degeneration and Vision Loss: AREDS Report No. 8. Arch. Ophthalmol..

[8] Chen Y., Vuong L.N., Liu J., Ho J., Srinivasan V.J., Gorczynska I., Witkin A.J., Duker J.S., Schuman J., Fujimoto J.G. (2003). Three-Dimensional Ultrahigh Resolution Optical Coherence Tomography Imaging of Age-Related Macular Degeneration. Opt. Express.

[9] Heesterbeek T.J., Lorés-Motta L., Hoyng C.B., Lechanteur Y.T.E., den Hollander A.I. (2020). Risk Factors for Progression of Age-Related Macular Degeneration. Ophthalmic Physiol. Opt..

[10] Shi Y., Zhang Q., Zhou H., Wang L., Chu Z., Jiang X., Shen M., Thulliez M., Lyu C., Feuer W. (2021). Correlations Between Choriocapillaris and Choroidal Measurements and the Growth of Geographic Atrophy Using Swept Source OCT Imaging. Am. J. Ophthalmol..

[11] Daien V., Finger R.P., Talks J.S., Mitchell P., Wong T.Y., Sakamoto T., Eldem B.M., Korobelnik J.-F. (2021). Evolution of Treatment Paradigms in Neovascular Age-Related Macular Degeneration: A Review of Real-World Evidence. Br. J. Ophthalmol..

[12] Lanzetta P., Korobelnik J.-F., Heier J.S., Leal S., Holz F.G., Lloyd Clark W., Eichenbaum D., Iida T., Xiaodong S., Berliner A.J. (2024). Intravitreal Aflibercept 8 Mg in Neovascular Age-Related Macular Degeneration (PULSAR): 48-Week Results from a Randomised, Double-Masked, Non-Inferiority, Phase 3 Trial. Lancet.

[13] Merle B.M.J., Rosner B., Seddon J.M. (2020). Genetic Susceptibility, Diet Quality, and Two-Step Progression in Drusen Size. Investig. Ophthalmol. Vis. Sci..

[14] Rowan S., Taylor A. (2016). Gene-Diet Interactions in Age-Related Macular Degeneration. Adv. Exp. Med. Biol..

[15] Colijn J.M., Meester-Smoor M., Verzijden T., de Breuk A., Silva R., Merle B.M.J., Cougnard-Grégoire A., Hoyng C.B., Fauser S., Coolen A. (2021). Genetic Risk, Lifestyle, and Age-Related Macular Degeneration in Europe: The EYE-RISK Consortium. Ophthalmology.

[16] Farinha C., Barreto P., Coimbra R., Machado M.B., Figueiredo I., Cachulo M.L., Cunha-Vaz J., Silva R. (2024). Age-Related Macular Degeneration and Extramacular Drusen: Genetic Associations in the Coimbra Eye Study. Investig. Ophthalmol. Vis. Sci..

[17] Fritsche L.G., Igl W., Bailey J.N.C., Grassmann F., Sengupta S., Bragg-Gresham J.L., Burdon K.P., Hebbring S.J., Wen C., Gorski M. (2016). A Large Genome-Wide Association Study of Age-Related Macular Degeneration Highlights Contributions of Rare and Common Variants. Nat. Genet..

[18] Geerlings M.J., de Jong E.K., den Hollander A.I. (2017). The Complement System in Age-Related Macular Degeneration: A Review of Rare Genetic Variants and Implications for Personalized Treatment. Mol. Immunol..

[19] Farinha C., Barreto P., Coimbra R., Cachulo M.L., Melo J.B., Cunha-Vaz J., Lechanteur Y., Hoyng C.B., Silva R. (2023). Common and Rare Genetic Risk Variants in Age-Related Macular Degeneration and Genetic Risk Score in the Coimbra Eye Study. Acta Ophthalmol..

[20] Hu M.L., Quinn J., Xue K. (2021). Interactions between Apolipoprotein E Metabolism and Retinal Inflammation in Age-Related Macular Degeneration. Life.

[21] Raimundo M., Mira F., Cachulo M.d.L., Barreto P., Ribeiro L., Farinha C., Laíns I., Nunes S., Alves D., Figueira J. (2018). Adherence to a Mediterranean Diet, Lifestyle and Age-Related Macular Degeneration: The Coimbra Eye Study—Report 3. Acta Ophthalmol..

[22] Klein R., Klein B.E.K., Tomany S.C., Cruickshanks K.J. (2003). The Association of Cardiovascular Disease with the Long-Term Incidence of Age-Related Maculopathy: The Beaver Dam Eye Study. Ophthalmology.

[23] Williams M.A., McKay G.J., Chakravarthy U. (2014). Complement Inhibitors for Age-Related Macular Degeneration. Cochrane Database Syst. Rev..

[24] Buch H., Vinding T., la Cour M., Jensen G.B., Prause J.U., Nielsen N.V. (2005). Risk Factors for Age-Related Maculopathy in a 14-Year Follow-up Study: The Copenhagen City Eye Study: Acta Ophthalmologica Scandinavica 2005. Acta Ophthalmol. Scand..

[25] Meyers K.J., Liu Z., Millen A.E., Iyengar S.K., Blodi B.A., Johnson E., Snodderly D.M., Klein M.L., Gehrs K.M., Tinker L. (2015). Joint Associations of Diet, Lifestyle, and Genes with Age-Related Macular Degeneration. Ophthalmology.

[26] Tomany S.C., Wang J.J., Van Leeuwen R., Klein R., Mitchell P., Vingerling J.R., Klein B.E.K., Smith W., De Jong P.T.V.M. (2004). Risk Factors for Incident Age-Related Macular Degeneration: Pooled Findings from 3 Continents. Ophthalmology.

[27] Altay L., Subiras X., Lorés de Motta L., Schick T., Berghold A., Hoyng C.B., den Hollander A.I., Fauser S., Sadda S.R., Liakopoulos S. (2020). Genetic and Environmental Risk Factors for Extramacular Drusen. Mol. Vis..

[28] Cachulo M.d.L., Laíns I., Lobo C., Figueira J., Ribeiro L., Marques J.P., Costa J., Vieira A., Rodrigues J., Alves D. (2016). Age-Related Macular Degeneration in Portugal: Prevalence and Risk Factors in a Coastal and an Inland Town. The Coimbra Eye Study—Report 2. Acta Ophthalmol..

[29] Rondanelli M., Gasparri C., Barrile G.C., Battaglia S., Cavioni A., Giusti R., Mansueto F., Moroni A., Nannipieri F., Patelli Z. (2022). Effectiveness of a Novel Food Composed of Leucine, Omega-3 Fatty Acids and Probiotic Lactobacillus Paracasei PS23 for the Treatment of Sarcopenia in Elderly Subjects: A 2-Month Randomized Double-Blind Placebo-Controlled Trial. Nutrients.

[30] Wu Y., Xie Y., Yuan Y., Xiong R., Hu Y., Ning K., Ha J., Wang W., Han X., He M. (2023). The Mediterranean Diet and Age-Related Eye Diseases: A Systematic Review. Nutrients.

[31] The Age-Related Eye Disease Study 2 (AREDS2) Research Group (2013). Lutein + Zeaxanthin and Omega-3 Fatty Acids for Age-Related Macular Degeneration: The Age-Related Eye Disease Study 2 (AREDS2) Randomized Clinical Trial. JAMA.

[32] Chew E.Y., Clemons T.E., Agrón E., Domalpally A., Keenan T.D.L., Vitale S., Weber C., Smith D.C., Christen W., AREDS2 Research Group (2022). Long-Term Outcomes of Adding Lutein/Zeaxanthin and ω-3 Fatty Acids to the AREDS Supplements on Age-Related Macular Degeneration Progression: AREDS2 Report 28: AREDS2 Report 28. JAMA Ophthalmol..

[33] Tuzcu M., Orhan C., Muz O.E., Sahin N., Juturu V., Sahin K. (2017). Lutein and Zeaxanthin Isomers Modulates Lipid Metabolism and the Inflammatory State of Retina in Obesity-Induced High-Fat Diet Rodent Model. BMC Ophthalmol..

[34] Chapman N.A., Jacobs R.J., Braakhuis A.J. (2019). Role of Diet and Food Intake in Age-Related Macular Degeneration: A Systematic Review. Clin. Experiment. Ophthalmol..

[35] Smailhodzic D., van Asten F., Blom A.M., Mohlin F.C., den Hollander A.I., van de Ven J.P.H., van Huet R.A.C., Groenewoud J.M.M., Tian Y., Berendschot T.T.J.M. (2014). Zinc Supplementation Inhibits Complement Activation in Age-Related Macular Degeneration. PLoS ONE.

[36] Weikel K.A., Fitzgerald P., Shang F., Caceres M.A., Bian Q., Handa J.T., Stitt A.W., Taylor A. (2012). Natural History of Age-Related Retinal Lesions That Precede AMD in Mice Fed High or Low Glycemic Index Diets. Investig. Ophthalmol. Vis. Sci..

[37] Rowan S., Weikel K., Chang M.-L., Nagel B.A., Thinschmidt J.S., Carey A., Grant M.B., Fliesler S.J., Smith D., Taylor A. (2014). Cfh Genotype Interacts with Dietary Glycemic Index to Modulate Age-Related Macular Degeneration-like Features in Mice. Investig. Ophthalmol. Vis. Sci..

[38] Seddon J.M., Ajani U.A., Sperduto R.D., Hiller R., Blair N., Burton T.C., Farber M.D., Gragoudas E.S., Haller J., Miller D.T. (1994). Dietary Carotenoids, Vitamins A, C, and E, and Advanced Age-Related Macular Degeneration. Eye Disease Case-Control Study Group. JAMA.

[39] SanGiovanni J.P., Chew E.Y., Clemons T.E., Davis M.D., Ferris F.L., Gensler G.R., Kurinij N., Lindblad A.S., Milton R.C., Seddon J.M. (2007). The Relationship of Dietary Lipid Intake and Age-Related Macular Degeneration in a Case-Control Study: AREDS Report No. 20. Arch. Ophthalmol..

[40] Tan J.S.L., Wang J.J., Flood V., Mitchell P. (2009). Dietary Fatty Acids and the 10-Year Incidence of Age-Related Macular Degeneration: The Blue Mountains Eye Study: The Blue Mountains Eye Study. Arch. Ophthalmol..

[41] Nani A., Murtaza B., Sayed Khan A., Khan N.A., Hichami A. (2021). Antioxidant and Anti-Inflammatory Potential of Polyphenols Contained in Mediterranean Diet in Obesity: Molecular Mechanisms. Molecules.

[42] Chong E.W.-T., Kreis A.J., Wong T.Y., Simpson J.A., Guymer R.H. (2008). Dietary Omega-3 Fatty Acid and Fish Intake in the Primary Prevention of Age-Related Macular Degeneration: A Systematic Review and Meta-Analysis: A Systematic Review and Meta-Analysis. Arch. Ophthalmol..

[43] Singh U., Devaraj S., Jialal I. (2005). Vitamin E, Oxidative Stress, and Inflammation. Annu. Rev. Nutr..

[44] Donoso L.A., Kim D., Frost A., Callahan A., Hageman G. (2006). The Role of Inflammation in the Pathogenesis of Age-Related Macular Degeneration. Surv. Ophthalmol..

[45] Yan Q., Ding Y., Liu Y., Sun T., Fritsche L.G., Clemons T., Ratnapriya R., Klein M.L., Cook R.J., Liu Y. (2018). Genome-Wide Analysis of Disease Progression in Age-Related Macular Degeneration. Hum. Mol. Genet..

[46] Yu Y., Reynolds R., Rosner B., Daly M.J., Seddon J.M. (2012). Prospective Assessment of Genetic Effects on Progression to Different Stages of Age-Related Macular Degeneration Using Multistate Markov Models. Investig. Ophthalmol. Vis. Sci..

[47] Kersten E., Paun C.C., Schellevis R.L., Hoyng C.B., Delcourt C., Lengyel I., Peto T., Ueffing M., Klaver C.C.W., Dammeier S. (2018). Systemic and Ocular Fluid Compounds as Potential Biomarkers in Age-Related Macular Degeneration. Surv. Ophthalmol..

[48] Fleckenstein M., Keenan T.D.L., Guymer R.H., Chakravarthy U., Schmitz-Valckenberg S., Klaver C.C., Wong W.T., Chew E.Y. (2021). Age-Related Macular Degeneration. Nat. Rev. Dis. Primers.

[49] Kortvely E., Hauck S.M., Behler J., Ho N., Ueffing M. (2016). The Unconventional Secretion of ARMS2. Hum. Mol. Genet..

[50] Canfield A.E., Hadfield K.D., Rock C.F., Wylie E.C., Wilkinson F.L. (2007). HtrA1: A Novel Regulator of Physiological and Pathological Matrix Mineralization?. Biochem. Soc. Trans..

[51] Beaufort N., Scharrer E., Kremmer E., Lux V., Ehrmann M., Huber R., Houlden H., Werring D., Haffner C., Dichgans M. (2014). Cerebral Small Vessel Disease-Related Protease HtrA1 Processes Latent TGF-β Binding Protein 1 and Facilitates TGF-β Signaling. Proc. Natl. Acad. Sci. USA.

[52] Grassmann F., Fleckenstein M., Chew E.Y., Strunz T., Schmitz-Valckenberg S., Göbel A.P., Klein M.L., Ratnapriya R., Swaroop A., Holz F.G. (2015). Clinical and Genetic Factors Associated with Progression of Geographic Atrophy Lesions in Age-Related Macular Degeneration. PLoS ONE.

[53] Miyake M., Yamashiro K., Tamura H., Kumagai K., Saito M., Sugahara-Kuroda M., Yoshikawa M., Oishi M., Akagi-Kurashige Y., Nakata I. (2015). The Contribution of Genetic Architecture to the 10-Year Incidence of Age-Related Macular Degeneration in the Fellow Eye. Investig. Ophthalmol. Vis. Sci..

[54] van Leeuwen E.M., Emri E., Merle B.M.J., Colijn J.M., Kersten E., Cougnard-Gregoire A., Dammeier S., Meester-Smoor M., Pool F.M., de Jong E.K. (2018). A New Perspective on Lipid Research in Age-Related Macular Degeneration. Prog. Retin. Eye Res..

[55] Toops K.A., Tan L.X., Lakkaraju A. (2016). Apolipoprotein E Isoforms and AMD. Adv. Exp. Med. Biol..

[56] Ocké M.C. (2013). Evaluation of Methodologies for Assessing the Overall Diet: Dietary Quality Scores and Dietary Pattern Analysis. Proc. Nutr. Soc..

[57] Rong S.S., Lee B.Y., Kuk A.K., Yu X.T., Li S.S., Li J., Guo Y., Yin Y., Osterbur D.L., Yam J.C.S. (2019). Comorbidity of Dementia and Age-Related Macular Degeneration Calls for Clinical Awareness: A Meta-Analysis. Br. J. Ophthalmol..

[58] Tan J.S.L., Wang J.J., Liew G., Rochtchina E., Mitchell P. (2008). Age-Related Macular Degeneration and Mortality from Cardiovascular Disease or Stroke. Br. J. Ophthalmol..

[59] Ozawa Y. (2020). Oxidative Stress in the Light-Exposed Retina and Its Implication in Age-Related Macular Degeneration. Redox Biol..

[60] Gonzalez-Iglesias H., Alvarez L., García M., Petrash C., Sanz-Medel A., Coca-Prados M. (2014). Metallothioneins (MTs) in the Human Eye: A Perspective Article on the Zinc-MT Redox Cycle. Metallomics.

[61] King J.C., Brown K.H., Gibson R.S., Krebs N.F., Lowe N.M., Siekmann J.H., Raiten D.J. (2015). Biomarkers of Nutrition for Development (BOND)-Zinc Review. J. Nutr..

[62] Tan J.S.L., Wang J.J., Flood V., Rochtchina E., Smith W., Mitchell P. (2008). Dietary Antioxidants and the Long-Term Incidence of Age-Related Macular Degeneration: The Blue Mountains Eye Study. Ophthalmology.

[63] Klein M.L., Francis P.J., Rosner B., Reynolds R., Hamon S.C., Schultz D.W., Ott J., Seddon J.M. (2008). CFH and LOC387715/ARMS2 Genotypes and Treatment with Antioxidants and Zinc for Age-Related Macular Degeneration. Ophthalmology.

[64] Ho L., van Leeuwen R., Witteman J.C.M., van Duijn C.M., Uitterlinden A.G., Hofman A., de Jong P.T.V.M., Vingerling J.R., Klaver C.C.W. (2011). Reducing the Genetic Risk of Age-Related Macular Degeneration with Dietary Antioxidants, Zinc, and ω-3 Fatty Acids: The Rotterdam Study: The Rotterdam Study. Arch. Ophthalmol..

[65] Awh C.C., Lane A.-M., Hawken S., Zanke B., Kim I.K. (2013). CFH and ARMS2 Genetic Polymorphisms Predict Response to Antioxidants and Zinc in Patients with Age-Related Macular Degeneration. Ophthalmology.

[66] Li Z.Y., Tso M.O., Wang H.M., Organisciak D.T. (1985). Amelioration of Photic Injury in Rat Retina by Ascorbic Acid: A Histopathologic Study. Investig. Ophthalmol. Vis. Sci..

[67] Braakhuis A., Raman R., Vaghefi E. (2017). The Association between Dietary Intake of Antioxidants and Ocular Disease. Diseases.

[68] Aoki A., Inoue M., Nguyen E., Obata R., Kadonosono K., Shinkai S., Hashimoto H., Sasaki S., Yanagi Y. (2016). Dietary N-3 Fatty Acid, α-Tocopherol, Zinc, Vitamin D, Vitamin C, and β-Carotene Are Associated with Age-Related Macular Degeneration in Japan. Sci. Rep..

[69] Sant D.W., Camarena V., Mustafi S., Li Y., Wilkes Z., Van Booven D., Wen R., Wang G. (2018). Ascorbate Suppresses VEGF Expression in Retinal Pigment Epithelial Cells. Investig. Ophthalmol. Vis. Sci..

[70] Tahiliani M., Koh K.P., Shen Y., Pastor W.A., Bandukwala H., Brudno Y., Agarwal S., Iyer L.M., Liu D.R., Aravind L. (2009). Conversion of 5-Methylcytosine to 5-Hydroxymethylcytosine in Mammalian DNA by MLL Partner TET1. Science.

[71] Young J.I., Züchner S., Wang G. (2015). Regulation of the Epigenome by Vitamin C. Annu. Rev. Nutr..

[72] West S., Vitale S., Hallfrisch J., Muñoz B., Muller D., Bressler S., Bressler N.M. (1994). Are Antioxidants or Supplements Protective for Age-Related Macular Degeneration?. Arch. Ophthalmol..

[73] Bendik I., Friedel A., Roos F.F., Weber P., Eggersdorfer M. (2014). Vitamin D: A Critical and Essential Micronutrient for Human Health. Front. Physiol..

[74] McKay G.J., Young I.S., McGinty A., Bentham G.C.G., Chakravarthy U., Rahu M., Seland J., Soubrane G., Tomazzoli L., Topouzis F. (2017). Associations between Serum Vitamin D and Genetic Variants in Vitamin D Pathways and Age-Related Macular Degeneration in the European Eye Study. Ophthalmology.

[75] Del Valle H.B., Yaktine A.L., Taylor C.L., Ross A.C., Institute of Medicine, Food and Nutrition Board, Committee to Review Dietary Reference Intakes for Vitamin D and Calcium (2011). Dietary Reference Intakes for Calcium and Vitamin D.

[76] Layana A.G., Minnella A.M., Garhöfer G., Aslam T., Holz F.G., Leys A., Silva R., Delcourt C., Souied E., Seddon J.M. (2017). Vitamin D and Age-Related Macular Degeneration. Nutrients.

[77] Millen A.E., Meyers K.J., Liu Z., Engelman C.D., Wallace R.B., LeBlanc E.S., Tinker L.F., Iyengar S.K., Robinson J.G., Sarto G.E. (2015). Association between Vitamin D Status and Age-Related Macular Degeneration by Genetic Risk. JAMA Ophthalmol..

[78] Millen A.E., Voland R., Sondel S.A., Parekh N., Horst R.L., Wallace R.B., Hageman G.S., Chappell R., Blodi B.A., Klein M.L. (2011). Vitamin D Status and Early Age-Related Macular Degeneration in Postmenopausal Women. Arch. Ophthalmol..

[79] Parekh N., Chappell R.J., Millen A.E., Albert D.M., Mares J.A. (2007). Association between Vitamin D and Age-Related Macular Degeneration in the Third National Health and Nutrition Examination Survey, 1988 through 1994. Arch. Ophthalmol..

[80] Cougnard-Grégoire A., Merle B.M.J., Korobelnik J.-F., Rougier M.-B., Delyfer M.-N., Féart C., Le Goff M., Dartigues J.-F., Barberger-Gateau P., Delcourt C. (2015). Vitamin D Deficiency in Community-Dwelling Elderly Is Not Associated with Age-Related Macular Degeneration. J. Nutr..

[81] Flood V., Smith W., Wang J.J., Manzi F., Webb K., Mitchell P. (2002). Dietary Antioxidant Intake and Incidence of Early Age-Related Maculopathy: The Blue Mountains Eye Study. Ophthalmology.

[82] Borel P. (2012). Genetic Variations Involved in Interindividual Variability in Carotenoid Status. Mol. Nutr. Food Res..

[83] Albanes D., Heinonen O.P., Taylor P.R., Virtamo J., Edwards B.K., Rautalahti M., Hartman A.M., Palmgren J., Freedman L.S., Haapakoski J. (1996). Alpha-Tocopherol and Beta-Carotene Supplements and Lung Cancer Incidence in the Alpha-Tocopherol, Beta-Carotene Cancer Prevention Study: Effects of Base-Line Characteristics and Study Compliance. J. Natl. Cancer Inst..

[84] Yonekura L., Nagao A. (2007). Intestinal Absorption of Dietary Carotenoids. Mol. Nutr. Food Res..

[85] Bartlett H., Howells O., Eperjesi F. (2010). The Role of Macular Pigment Assessment in Clinical Practice: A Review: Assessment of Macular Pigment. Clin. Exp. Optom..

[86] Korobelnik J.-F., Rougier M.-B., Delyfer M.-N., Bron A., Merle B.M.J., Savel H., Chêne G., Delcourt C., Creuzot-Garcher C. (2017). Effect of Dietary Supplementation with Lutein, Zeaxanthin, and ω-3 on Macular Pigment: A Randomized Clinical Trial. JAMA Ophthalmol..

[87] Conrady C.D., Bell J.P., Besch B.M., Gorusupudi A., Farnsworth K., Ermakov I., Sharifzadeh M., Ermakova M., Gellermann W., Bernstein P.S. (2017). Correlations between Macular, Skin, and Serum Carotenoids. Investig. Ophthalmol. Vis. Sci..

[88] Ma L., Liu R., Du J.H., Liu T., Wu S.S., Liu X.H. (2016). Lutein, Zeaxanthin and Meso-Zeaxanthin Supplementation Associated with Macular Pigment Optical Density. Nutrients.

[89] Robman L., Vu H., Hodge A., Tikellis G., Dimitrov P., McCarty C., Guymer R. (2007). Dietary Lutein, Zeaxanthin, and Fats and the Progression of Age-Related Macular Degeneration. Can. J. Ophthalmol..

[90] Keenan T.D.L., Agrón E., Keane P.A., Domalpally A., Chew E.Y., Age-Related Eye Disease Study Research Group (2024). Age-Related Eye Disease Study 2 Research Group Oral Antioxidant and Lutein/Zeaxanthin Supplements Slow Geographic Atrophy Progression to the Fovea in Age-Related Macular Degeneration. Ophthalmology.

[91] Hammond C.J., Liew S.H.M., Van Kuijk F.J., Beatty S., Nolan J.M., Spector T.D., Gilbert C.E. (2012). The Heritability of Macular Response to Supplemental Lutein and Zeaxanthin: A Classic Twin Study. Investig. Ophthalmol. Vis. Sci..

[92] Ma L., Yan S.-F., Huang Y.-M., Lu X.-R., Qian F., Pang H.-L., Xu X.-R., Zou Z.-Y., Dong P.-C., Xiao X. (2012). Effect of Lutein and Zeaxanthin on Macular Pigment and Visual Function in Patients with Early Age-Related Macular Degeneration. Ophthalmology.

[93] SanGiovanni J.P., Neuringer M. (2012). The Putative Role of Lutein and Zeaxanthin as Protective Agents against Age-Related Macular Degeneration: Promise of Molecular Genetics for Guiding Mechanistic and Translational Research in the Field. Am. J. Clin. Nutr..

[94] Ozawa Y., Sasaki M., Takahashi N., Kamoshita M., Miyake S., Tsubota K. (2012). Neuroprotective Effects of Lutein in the Retina. Curr. Pharm. Des..

[95] Park S.W., Cho C.S., Jun H.O., Ryu N.H., Kim J.H., Yu Y.S., Kim J.S., Kim J.H. (2012). Anti-Angiogenic Effect of Luteolin on Retinal Neovascularization via Blockade of Reactive Oxygen Species Production. Investig. Ophthalmol. Vis. Sci..

[96] Meyers K.J., Mares J.A., Igo R.P., Truitt B., Liu Z., Millen A.E., Klein M., Johnson E.J., Engelman C.D., Karki C.K. (2014). Genetic Evidence for Role of Carotenoids in Age-Related Macular Degeneration in the Carotenoids in Age-Related Eye Disease Study (CAREDS). Investig. Ophthalmol. Vis. Sci..

[97] Tan J.S.L., Mitchell P., Smith W., Wang J.J. (2007). Cardiovascular Risk Factors and the Long-Term Incidence of Age-Related Macular Degeneration: The Blue Mountains Eye Study. Ophthalmology.

[98] Yip J.L.Y., Khawaja A.P., Chan M.P.Y., Broadway D.C., Peto T., Tufail A., Luben R., Hayat S., Bhaniani A., Wareham N.J. (2015). Cross Sectional and Longitudinal Associations between Cardiovascular Risk Factors and Age Related Macular Degeneration in the EPIC-Norfolk Eye Study. PLoS ONE.

[99] Saunier V., Merle B.M.J., Delyfer M.-N., Cougnard-Grégoire A., Rougier M.-B., Amouyel P., Lambert J.-C., Dartigues J.-F., Korobelnik J.-F., Delcourt C. (2018). Incidence of and Risk Factors Associated with Age-Related Macular Degeneration: Four-Year Follow-up from the ALIENOR Study. JAMA Ophthalmol..

[100] Ngai L.-Y., Stocks N., Sparrow J.M., Patel R., Rumley A., Lowe G., Smith G.D., Ben-Shlomo Y. (2011). The Prevalence and Analysis of Risk Factors for Age-Related Macular Degeneration: 18-Year Follow-up Data from the Speedwell Eye Study, United Kingdom. EYE.

[101] Reynolds R., Rosner B., Seddon J.M. (2013). Dietary Omega-3 Fatty Acids, Other Fat Intake, Genetic Susceptibility, and Progression to Incident Geographic Atrophy. Ophthalmology.

[102] Merle B.M.J., Buaud B., Korobelnik J.-F., Bron A., Delyfer M.-N., Rougier M.-B., Savel H., Vaysse C., Creuzot-Garcher C., Delcourt C. (2017). Plasma Long-Chain Omega-3 Polyunsaturated Fatty Acids and Macular Pigment in Subjects with Family History of Age-Related Macular Degeneration: The Limpia Study. Acta Ophthalmol..

[103] Parekh N., Voland R.P., Moeller S.M., Blodi B.A., Ritenbaugh C., Chappell R.J., Wallace R.B., Mares J.A., CAREDS Research Study Group (2009). Association between Dietary Fat Intake and Age-Related Macular Degeneration in the Carotenoids in Age-Related Eye Disease Study (CAREDS): An Ancillary Study of the Women’s Health Initiative. Arch. Ophthalmol..

[104] Cougnard-Grégoire A., Merle B.M.J., Korobelnik J.-F., Rougier M.-B., Delyfer M.-N., Le Goff M., Samieri C., Dartigues J.-F., Delcourt C. (2016). Olive Oil Consumption and Age-Related Macular Degeneration: The Alienor Study. PLoS ONE.

[105] Arterburn L.M., Hall E.B., Oken H. (2006). Distribution, Interconversion, and Dose Response of n-3 Fatty Acids in Humans. Am. J. Clin. Nutr..

[106] Wu J., Cho E., Giovannucci E.L., Rosner B.A., Sastry S.M., Schaumberg D.A., Willett W.C. (2017). Dietary Intake of α-Linolenic Acid and Risk of Age-Related Macular Degeneration. Am. J. Clin. Nutr..

[107] Souied E., Delcourt C., Querques G., Merle B., Smith T., Benlian P. (2013). Nat2 Study: Omega-3 Levels in Red Blood Cells Membranes Correlates the Preventive Effect. Investig. Ophthalmol. Vis. Sci..

[108] Souied E.H., Aslam T., Garcia-Layana A., Holz F.G., Leys A., Silva R., Delcourt C. (2015). Omega-3 Fatty Acids and Age-Related Macular Degeneration. Ophthalmic Res..

[109] Simon E., Bardet B., Grégoire S., Acar N., Bron A.M., Creuzot-Garcher C.P., Bretillon L. (2011). Decreasing Dietary Linoleic Acid Promotes Long Chain Omega-3 Fatty Acid Incorporation into Rat Retina and Modifies Gene Expression. Exp. Eye Res..

[110] Anderson D.H., Mullins R.F., Hageman G.S., Johnson L.V. (2002). A Role for Local Inflammation in the Formation of Drusen in the Aging Eye. Am. J. Ophthalmol..

[111] Seddon J.M., Rosner B., Sperduto R.D., Yannuzzi L., Haller J.A., Blair N.P., Willett W. (2001). Dietary Fat and Risk for Advanced Age-Related Macular Degeneration. Arch. Ophthalmol..

[112] Hegele R.A. (2009). Plasma Lipoproteins: Genetic Influences and Clinical Implications. Nat. Rev. Genet..

[113] Wang Y., Wang M., Zhang X., Zhang Q., Nie J., Zhang M., Liu X., Ma L. (2016). The Association between the Lipids Levels in Blood and Risk of Age-Related Macular Degeneration. Nutrients.

[114] Burgess S., Davey Smith G. (2017). Mendelian Randomization Implicates High-Density Lipoprotein Cholesterol-Associated Mechanisms in Etiology of Age-Related Macular Degeneration. Ophthalmology.

[115] Eren E., Yilmaz N., Aydin O. (2012). High Density Lipoprotein and It’s Dysfunction. Open Biochem. J..

[116] Pertl L., Kern S., Weger M., Hausberger S., Trieb M., Gasser-Steiner V., Haas A., Scharnagl H., Heinemann A., Marsche G. (2016). High-Density Lipoprotein Function in Exudative Age-Related Macular Degeneration. PLoS ONE.

[117] Teslovich T.M., Musunuru K., Smith A.V., Edmondson A.C., Stylianou I.M., Koseki M., Pirruccello J.P., Ripatti S., Chasman D.I., Willer C.J. (2010). Biological, Clinical and Population Relevance of 95 Loci for Blood Lipids. Nature.

[118] Willer C.J., Schmidt E.M., Sengupta S., Peloso G.M., Gustafsson S., Kanoni S., Ganna A., Chen J., Buchkovich M.L., Mora S. (2013). Discovery and Refinement of Loci Associated with Lipid Levels. Nat. Genet..

[119] Abecasis G.R., Altshuler D., Auton A., Brooks L.D., Durbin R.M., Gibbs R.A., Hurles M.E., McVean G.A., 1000 Genomes Project Consortium (2010). A Map of Human Genome Variation from Population-Scale Sequencing. Nature.

[120] Peloso G.M., Auer P.L., Bis J.C., Voorman A., Morrison A.C., Stitziel N.O., Brody J.A., Khetarpal S.A., Crosby J.R., Fornage M. (2014). Association of Low-Frequency and Rare Coding-Sequence Variants with Blood Lipids and Coronary Heart Disease in 56,000 Whites and Blacks. Am. J. Hum. Genet..

[121] Guerra R., Wang J., Grundy S.M., Cohen J.C. (1997). A Hepatic Lipase (LIPC) Allele Associated with High Plasma Concentrations of High Density Lipoprotein Cholesterol. Proc. Natl. Acad. Sci. USA.

[122] Liutkeviciene R., Vilkeviciute A., Kriauciuniene L., Deltuva V.P. (2019). SIRT1 Rs12778366, FGFR2 Rs2981582, STAT3 Rs744166, LIPC Rs10468017, Rs493258 and LPL Rs12678919 Genotypes and Haplotype Evaluation in Patients with Age-Related Macular Degeneration. Gene.

[123] Wang Y.-F., Han Y., Zhang R., Qin L., Wang M.-X., Ma L. (2015). CETP/LPL/LIPC Gene Polymorphisms and Susceptibility to Age-Related Macular Degeneration. Sci. Rep..

[124] Shughoury A., Sevgi D.D., Ciulla T.A. (2022). Molecular Genetic Mechanisms in Age-Related Macular Degeneration. Genes.

[125] Neale B.M., Fagerness J., Reynolds R., Sobrin L., Parker M., Raychaudhuri S., Tan P.L., Oh E.C., Merriam J.E., Souied E. (2010). Genome-Wide Association Study of Advanced Age-Related Macular Degeneration Identifies a Role of the Hepatic Lipase Gene (LIPC). Proc. Natl. Acad. Sci. USA.

[126] Sharma K., Battu P., Singh R., Sharma S.K., Anand A. (2022). Modulated Anti-VEGF Therapy under the Influence of Lipid Metabolizing Proteins in Age Related Macular Degeneration: A Pilot Study. Sci. Rep..

[127] Malek G., Johnson L.V., Mace B.E., Saloupis P., Schmechel D.E., Rickman D.W., Toth C.A., Sullivan P.M., Bowes Rickman C. (2005). Apolipoprotein E Allele-Dependent Pathogenesis: A Model for Age-Related Retinal Degeneration. Proc. Natl. Acad. Sci. USA.

[128] Storti F., Raphael G., Griesser V., Klee K., Drawnel F., Willburger C., Scholz R., Langmann T., von Eckardstein A., Fingerle J. (2017). Regulated Efflux of Photoreceptor Outer Segment-Derived Cholesterol by Human RPE Cells. Exp. Eye Res..

[129] Tall A.R., Rader D.J. (2018). Trials and Tribulations of CETP Inhibitors. Circ. Res..

[130] Rudolf M., Mir Mohi Sefat A., Miura Y., Tura A., Raasch W., Ranjbar M., Grisanti S., Aherrahrou Z., Wagner A., Messinger J.D. (2018). ApoA-I Mimetic Peptide 4F Reduces Age-Related Lipid Deposition in Murine Bruch’s Membrane and Causes Its Structural Remodeling. Curr. Eye Res..

[131] Reddy S.T., Navab M., Anantharamaiah G.M., Fogelman A.M. (2014). Apolipoprotein A-I Mimetics. Curr. Opin. Lipidol..

[132] Watson C.E., Weissbach N., Kjems L., Ayalasomayajula S., Zhang Y., Chang I., Navab M., Hama S., Hough G., Reddy S.T. (2011). Treatment of Patients with Cardiovascular Disease with L-4F, an Apo-A1 Mimetic, Did Not Improve Select Biomarkers of HDL Function. J. Lipid Res..

[133] Huang C., Xu Y., Li X., Wang W. (2013). Vascular Endothelial Growth Factor A Polymorphisms and Age-Related Macular Degeneration: A Systematic Review and Meta-Analysis. Mol. Vis..

[134] Samra Y.A., Zaidi Y., Rajpurohit P., Raghavan R., Cai L., Kaddour-Djebbar I., Tawfik A. (2023). Warburg Effect as a Novel Mechanism for Homocysteine-Induced Features of Age-Related Macular Degeneration. Int. J. Mol. Sci..

[135] Chen L., Qin Y., Liu B., Gao M., Li A., Li X., Gong G. (2022). PGC-1α-Mediated Mitochondrial Quality Control: Molecular Mechanisms and Implications for Heart Failure. Front. Cell Dev. Biol..

[136] Zhang M., Chu Y., Mowery J., Konkel B., Galli S., Theos A.C., Golestaneh N. (2018). Pgc-1α Repression and High-Fat Diet Induce Age-Related Macular Degeneration-like Phenotypes in Mice. Dis. Model. Mech..

[137] Zhou S., Taskintuna K., Hum J., Gulati J., Olaya S., Steinman J., Golestaneh N. (2024). PGC-1α Repression Dysregulates Lipid Metabolism and Induces Lipid Droplet Accumulation in Retinal Pigment Epithelium. Cell Death Dis..

[138] Fan Q., Maranville J.C., Fritsche L., Sim X., Cheung C.M.G., Chen L.J., Gorski M., Yamashiro K., Ahn J., Laude A. (2017). HDL-Cholesterol Levels and Risk of Age-Related Macular Degeneration: A Multiethnic Genetic Study Using Mendelian Randomization. Int. J. Epidemiol..

[139] Dao D., Xie B., Nadeem U., Xiao J., Movahedan A., D’Souza M., Leone V., Hariprasad S.M., Chang E.B., Sulakhe D. (2021). High-Fat Diet Alters the Retinal Transcriptome in the Absence of Gut Microbiota. Cells.

